# Seminal plasma induces inflammation in the uterus through the γδ T/IL-17 pathway

**DOI:** 10.1038/srep25118

**Published:** 2016-04-25

**Authors:** Zhi-Hui Song, Zhong-Yin Li, Dan-Dan Li, Wen-Ning Fang, Hai-Yan Liu, Dan-Dan Yang, Chao-Yang Meng, Ying Yang, Jing-Pian Peng

**Affiliations:** 1 State Key Laboratory of Stem Cell and Reproductive Biology, Institute of Zoology, Chinese Academy of Sciences, Beijing, P.R. China; 2 University of Chinese Academy of Sciences, Beijing, P.R. China

## Abstract

After insemination, a large number of leukocytes migrate into the uterus, which is accompanied by intense inflammation. However, the details of how seminal plasma interacts with the uterus are still not very clear. Here, we present that neutrophils migrate and accumulate around the uterine epithelium following insemination, which is accompanied by an increase in interleukin (IL) 17A levels. Additionally, we find that γδ T cells are the major source of IL-17A, and the seminal plasma could induce the γδ T cells to secret IL-17A. Blocking IL-17A could reduce the number of neutrophils in the uterus and prevent them from migrating to the epithelium by decreasing the chemokines CXCL1, CXCL2 and CXCL5. Blocking IL-17A did not affect the Th1/Th2 balance but actually diminished the inflammation in the uterus by reducing the expression of IL-1β and TNF-α. In summary, we found a new mechanism by which seminal plasma could influence the inflammation in the uterus through the γδ T/IL-17 pathway to regulate the expression of various chemokines and cytokines.

Recent research has demonstrated that seminal plasma can significantly improve implantation following *in vitro* fertilization (IVF)^1^, but the mechanism is not clear. Seminal plasma contains a variety of cytokines and growth factors, which can regulate inflammatory responses, leukocytes recruitment and the activation of innate and adaptive immunity^2^,^3^,^4^,^5^. Moderate inflammation can play an important role in a successful implantation^6^. Seminal plasma can also induce global changes in gene expression, which can affect cell migration, cell proliferation and cell viability^7^. Additionally, CD38 in seminal plasma can induce tolerogenic dendritic cells and regulatory T cells (Tregs)^4^. According to the literature, seminal plasma is not just a transport medium for spermatozoa but can also interact with the uterus to prepare the optimal environment for implantation^8^.

Interleukin (IL)-17A is a member of the IL-17 family, which includes IL-17A through IL-17F^9^. IL-17A has been shown to initiate a potent inflammatory response. In addition, IL-17 has been shown to regulate the expression of matrix metalloproteinases, cytokines and chemokines^10^,^11^,^12^,^13^. In addition to being a signature cytokine for T helper 17 (TH17) cells, IL-17A is also expressed by CD8^+^ T cells, γδ T cells, nature killer T cells and innate lymphoid cells^14^,^15^. In naive mice, γδ T cells constitute a minor subset of the cells in the blood and lymphoid tissue, but they perform functions similar to αβ T cells and play an important role in inflammation and tolerance^16^,^17^. Mouse Vγ6/Vδ1 cells are found to be closely associated with epithelial tissue in the female reproductive tract and account for the major proportion of γδ T cells in uterine tissue^18^,^19^,^20^. Unlike other γδ T cells, Vγ6/Vδ1 cells displayed a canonical Vg6 TCR amino acid junction^18^,^19^. This invariant subset could promote cancer growth through the γδ T/IL-17A/small peritoneal macrophages axis and protect against intestinal infection depending on the production of interferon gamma (IFN-γ) and IL-17A^21^,^22^.

It is now recognized that seminal plasma does not function solely as a transport and nutrient medium for spermatozoa^1^. In our work, we found that seminal plasma can stimulate γδ T cells to secret IL-17A, which regulates the secretion of cytokines (IL-1β and TNF-α) and chemokines (CXCL1, 2, 5 and CCL20). Chemokines then promote the recruitment of neutrophils to promote uterine inflammation.

## Results

### Neutrophils migrate into the uterus after insemination

**Figure 1:**
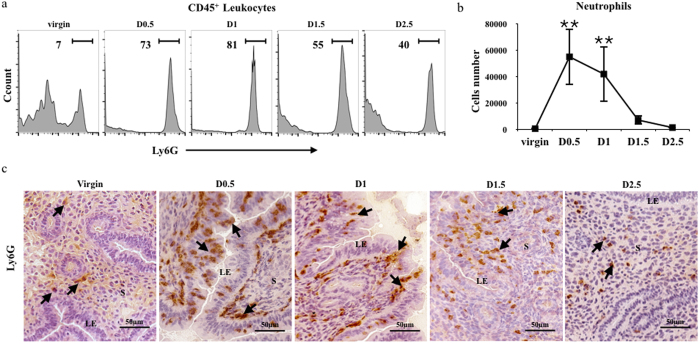
Neutrophils migrated into the uterus after insemination. (**a**) The percentage of neutrophils in the CD45^+^ population in the uterus was measured at the indicated days. The numbers represent the percentage of the population within the indicated gates. (**b**) Absolute numbers of neutrophils in the uterus were measured at the indicated days. The data are shown as the mean d S.E.M. from four independent experiments and independent t-tests. (**c**) Ly6G^+^ neutrophils in the uterus were analysed by immunohistochemistry at the indicated days. Scale bar: 500 μm. Arrows indicate Ly6G positive cells. The photomicrographs are representative of four mice from each group. LE, luminal epithelium; S, stroma.

The proportion of neutrophils among the CD45^**+**^ leukocyte population in the uterus was detected at different time points (virgin, D0.5 D1, D1.5 and D2.5) (Fig. 1a), and the Ly6G^**+**^ cells accounted for the majority of the cells in the uterus after insemination. Cell numbers were also counted at the same time points described above (Fig. 1b). Forty to seventy thousand neutrophils migrated into the uterus on D0.5 (P = 0.005) and D1 (P = 0.007), which significantly elevated the number of neutrophils in the uterus before quickly declining on D1.5 and D2.5. Histological analysis revealed (Fig. 1c) that neutrophils were barely detectable in the uterine stroma of virgin mice, but on D0.5 and D1 following implantation, a large number of neutrophils accumulated around the luminal epithelium and some of them even migrated into the uterine cavity. On D1.5, neutrophils were substantially elevated in the stroma, whereas very few neutrophils could be detected in the stroma one day later.

### Seminal plasma stimulated the γδ T cells to secrete IL-17A

**Figure 2:**
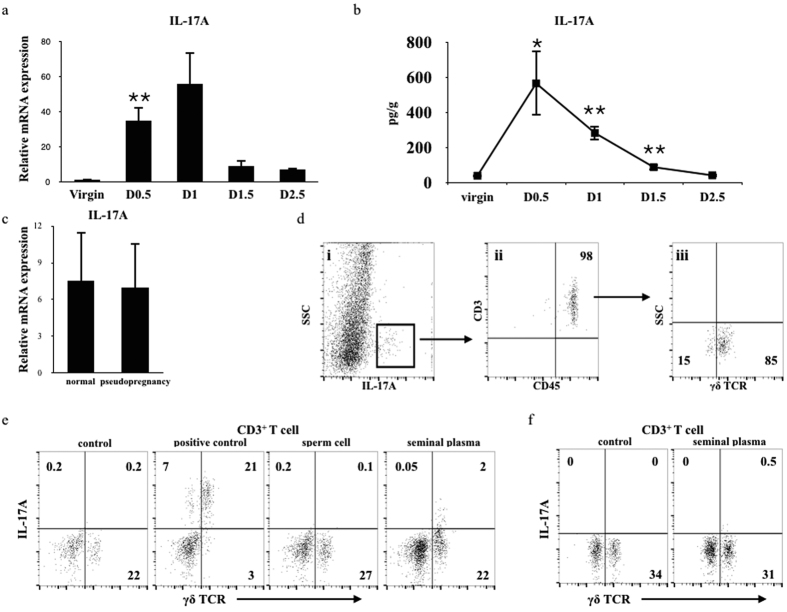
Sperm plasma induced the expression of IL-17A. (**a**) IL-17A expression was analysed by quantitative PCR in uteri at the indicated days. The data are shown as the mean ± S.E.M. from four independent experiments and independent t-tests. (**b**) IL-17A expression was analysed by ELISA in uteri at the indicated times. The data are shown as the mean ± S.E.M. from four independent experiments and independent t-tests. (**c**) IL-17A expression was analysed by quantitative PCR in uteri from female mice that were mated with normal or vasectomized males on D0.5. The data are shown as the mean ± S.E.M. from five independent experiments with independent t-tests. (**d**) Uterine cells from virgin mice were stimulated with the Cell Stimulation Cocktail (eBioscience) and analysed for CD3, γδ TCR, IL-17A and CD45 expression in four independent experiments. The numbers represent the percentage of the population within the indicated gates. i: whole uteri cells; ii: gated on the IL-17A positive population; iii: gated on the CD3^+^ CD45^+^ population. (**e**) Uterine cells from virgin mice were co-cultured with different stimulators (Control: brefeldin A; Positive control: 2 μl/mL of the Cell Stimulation Cocktail; Sperm cell: sperm cell (1:1) and brefeldin A; Seminal plasma: 0.5% seminal plasma and brefeldin A) as indicated and analysed for CD3, γδTCR, IL-17A and CD45 expression after stimulation in four independent experiments. The representative FACS profile was gated by CD3^+^ CD45^+^. (**f**) T cells sorted from mouse uterine tissue was labelled with CD3, co-cultured with the different stimulators as indicated above and analysed for γδTCR and IL-17A expression in three independent experiments. The numbers represent the percentage of the population within the indicated gates.

Given the pivotal roles of IL-17A in skin inflammation and neutrophil accumulation^23^,^24^, we tested the mRNA and protein expression of IL-17A at different time points (Fig. 2a,b). The mRNA expression of IL-17A was dramatically elevated after insemination on D0.5 (P = 0.002) and D1 (p = 0.06). In addition, the protein expression of IL-17A in uteri was also elevated on D0.5 (564 pg/g) (P = 0.011) and declined on D1 (281 pg/g) (P = 0.001) and D1.5 (87 pg/g) (P = 0.002). Using the pseudopregnancy model, we found that there was no difference in the mRNA expression of IL-17A between the pseudopregnancy and normal pregnancy models (Fig. 2c). To determine which cells secreted IL-17A, we tested a single suspension of cells from whole uterine tissue and gated on the IL-17A positive cells (Fig. 2d). We found that T cells predominantly expressed IL-17A. Additionally, we found that γδ T cells but not αβ T cells (CD4 and CD8) were the major source of IL-17A in the uterus. To detect the expression of IL-17A *in vitro*, we co-cultured the single suspension of cells derived from uterine tissue with different stimulators (Fig. 2e). Both the Cell Stimulation Cocktail (P = 0.0003) and seminal plasma (P = 0.041) but not the sperm cells significantly stimulated the γδ T cells to secrete IL-17A (Supplementary Fig. 1a). To determine whether other cells participated in the stimulation, we sorted the T cells from the uteri of virgin mice and co-cultured the cells with seminal plasma. We found that the T cells alone could also be stimulated by the seminal but not significantly (Fig. 2f and Supplementary Fig. 1b); there was no significant different from whole uterine single cells (Supplementary Fig. 1c).

### Neutralizing IL-17A attenuates neutrophil migration

**Figure 3:**
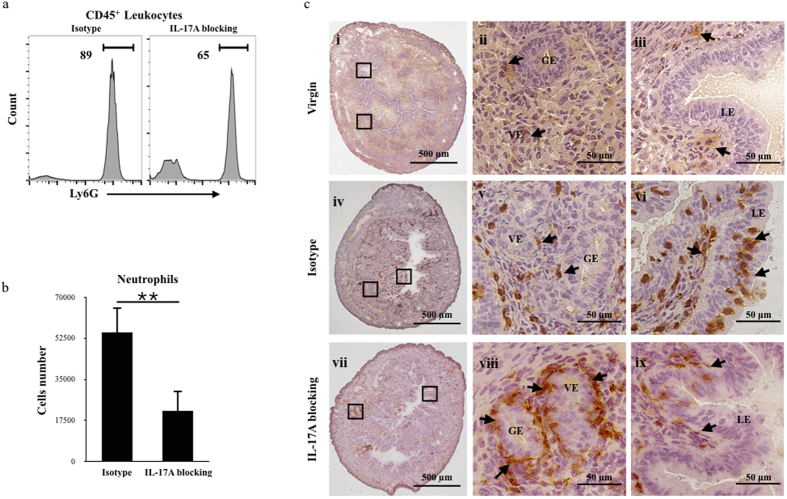
IL-17A neutralization disturbed the migration of neutrophils. (**a**) The percentage of neutrophils in the CD45-gated population in the uterus was measured at D0.5. The numbers represent the percentage of the population within the indicated gates. (**b**) Absolute numbers of neutrophils in the uterus were measured at D0.5. The data are shown as the mean ± S.E.M. from four (Isotype) or eight (IL-17A blocking) independent experiments and independent t-tests. (**c**) Ly6G stained neutrophils in the uterus were analysed by immunohistochemistry at D1 and virgin. The arrow indicates Ly6G positive cells. The photomicrographs are representative of three mice in each group. Panels ii, iii, v, vi and viii, ix are higher magnifications of different areas marked by the black rectangles in panels i, iv and vii. LE, luminal epithelium; S, stroma, VE, vessels epithelium; GE, gland epithelium. Scale bar: 500 μm (I, iv and vii) and 50 μm (ii, iii, v, vi and viii, ix).

To confirm whether IL-17A regulates the migration of neutrophils, we treated the mice intravenously with an IL-17A-neutralizing or an isotype-matched antibody (200 μg/kg) on D0.5. The proportion of neutrophils among the CD45^**+**^ leukocytes in the uterus was detected on D1 (Fig. 3a), and the numbers of neutrophils were counted at the same time (Fig. 3b). The percentage of neutrophils dropped from 89% to 65% and the cell number significantly declined (P = 0.0002) from fifty thousand to twenty thousand following treatment with an IL-17A-neutralizing antibody. Importantly, there was no change in the blood or spleen of mice following treatment with an IL-17A-neutralizing antibody (Supplementary Fig. 2). Immunohistochemical analysis demonstrated that the migration of neutrophils was disturbed after IL-17A blocking (Fig. 3c), with fewer neutrophils being distributed around the luminal epithelium. However, more neutrophils accumulated in the nearby vascular and gland epithelia.

### IL-17A regulates the expression of chemokines and cytokines in the uterus

**Figure 4:**
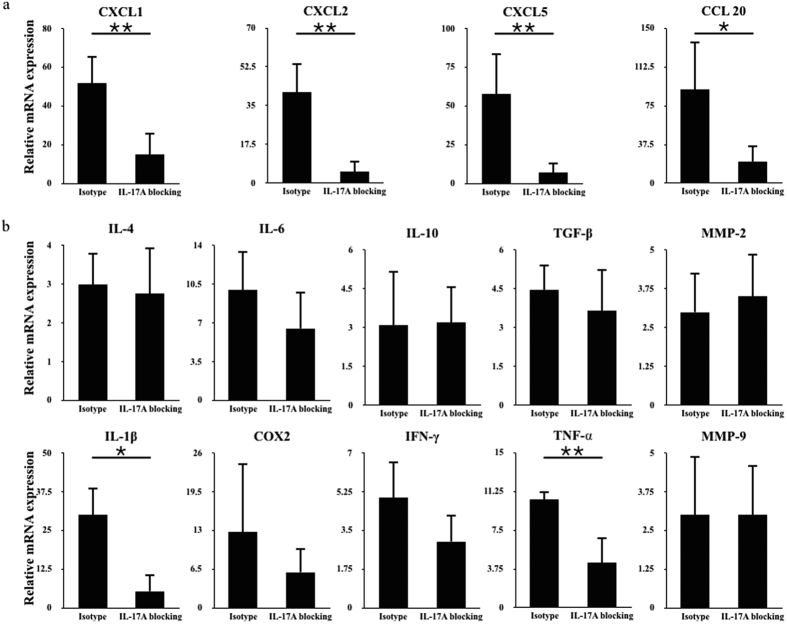
IL-17A blocking influenced the expression of chemokines and cytokines in the uterus. (**a**) The expression of chemokines was analysed by quantitative PCR in the uteri after IL-17A blocking. The data are shown as the mean ± S.E.M. from four independent experiments and independent t-tests. (**b**) The expression of cytokines and metalloproteinases was analysed by quantitative PCR in the uteri after IL-17A blocking. The data are shown as the mean ± S.E.M. from four independent experiments and independent t-tests.

To identify the mechanism by which neutrophils are recruited, we examined the chemokines related to neutrophil migration that could be regulated by IL-17A^25^,^26^ (Fig. 4a). All the chemokines (CXCL1, P = 0.008; CXCL2, P = 0.001; CXCL5, P = 0.005; CCL20, P = 0.019) that were evaluated were significantly downregulated after IL-17A neutralization. Specifically, CXCL2 and CXCL5 were reduced to one-eighth of their original levels. In addition, we examined the cytokines related to the microenvironment of the uterus (Fig. 4b). Only IL-1b (P = 0.027) and TNF-α (P = 0.003) were significantly reduced, and both of them have a pivotal role in promoting inflammation.

## Discussion

Insemination can lead to inflammation and neutrophil migration into the uterus^27^,^28^,^29^. In mice, a striking infiltration of neutrophils was observed in the uterus^28^, but the specific process by which this occurred was not yet clear. Here, we show that insemination induces an increase in the number of neutrophils in the uterus with the majority of the neutrophils being distributed around the luminal epithelium and a few entering the uterine cavity, which demonstrated that neutrophils may interact with sperm cells or promote inflammation^30^. This work supports previous work reported by SA Robertson^28^. Although the number of neutrophils in the uterus significantly increased immediately after insemination, the number rapidly declined on D1.5 and neutrophils were rarely found in the stroma on D2.5. This rapid and intense process may be important to ensure an optimal environment in the uterus for successful implantation^31^.

IL-17A plays an important role in skin inflammation and neutrophil migration^23^,^24^. Although many subsets of immune cells can secret IL-17A, such as CD8^+^ T cells (TC17), CD4^+^ T cells (Th17), γδ T cells (γδ T17) and NKT cells^13^, the dominant IL-17A expressing cells in the uterus were the γδ T cells. γδ T cells have been conserved for over 450 million years. The TCR of γδ T cells are more limited than αβ T cells, and thus, they can recognize only a limited number of antigens. Vγ6/Vδ1 T cells are rare in most tissues but are predominantly found in mouse uterine tissue^32^,^33^. Considering that γδ T cells are conserved and have a very specific distribution pattern, it is likely that the process by which seminal plasma induces the γδ T cells to secrete IL-17A to prepare the uterus for implantation is evolutionarily conserved in viviparous animals. Although a prior study has shown that testicular cells could stimulated the response of Vγ6/Vδ1 T cells^34^, we found that sperm cells alone were unable to induce γδ T cells to secrete IL-17A. TGF-β is abundant in seminal plasma and may play a key role in the generation γδ T17 cells^8^,^35^. It is known that Vγ6/Vδ1 cells have a canonical TCR, but the antigens recognized by Vγ6/Vδ1 T cells are still not clear^23^. Therefore, it is possible that Vγ6/Vδ1 cells could be stimulated by a specific antigen.

Seminal plasma also contains IL-17A, but the concentration of IL-17A is lower in seminal plasma (about 10 pg/ml) than serum (about 180 pg/ml) under normal conditions^2^,^36^. However, because insemination leads to a rapid increase in IL-17A expression, the neutralizing antibody may function primarily by neutralizing endogenous IL-17A. After IL-17A is blocked, the number of neutrophils decreases dramatically. IL-17A may induce the luminal epithelium or stroma to secrete chemokines to recruit neutrophils. Vγ6/Vδ1 T cells are closely associated with the epithelium^18^. Therefore, secreted IL-17A can interact with the epithelium quickly, which may explain why neutrophil migration to the epithelium was prevented after IL-17A blocking. While IL-17A is not the only pathway for insemination induced neutrophil recruitment, prior evidence showed that seminal plasma could stimulate the epithelial cells and stromal cells to express CXCL1 or CXCL2^7^. However, the recruitment of neutrophils is not the only function of IL-17A. Specifically, IL-17A has complex pro-inflammatory functions and can act on a broad range of cell types^23^.

Because IL-17A can induce the expression of cytokines, chemokines and metalloproteinases^10^,^11^,^12^,^13^, we tested the expression of related genes to evaluate the changes of the microenvironment in the uterus following the neutralization of IL-17A. First, we found that the expression of all the chemokines we evaluated was significantly decreased. CCL20, the ligand for CCR6, can be induced by IL-17A directly^37^ to promote inflammation and regulate both immune tolerance and activation. In addition to CXCL1 (ligand for CXCR1), CXCL2 and CXCL5 (ligand for CXCR2) are the primary chemokines induced for neutrophils recruitment^38^. Among these, the expression of CXCL2 and CXCL5 decreased dramatically. This may represent the process whereby the recruitment of neutrophils is primarily dependent on the CXCR2 pathway, which is similar to the migration of neutrophils into the lymph node during inflammation^39^. Second, we checked the genes related to the microenvironment of the uterus^40^,^41^,^42^ and found that only IL-1β and TNF-α, which promote inflammation^43^, decreased dramatically. Both vasectomized and normal male mouse could iαnduce the expression of TNF-α and IL-1β in uteri on D0.5, but mechanical stimulation failed^44^. The Th1 (IFN-γ) and Th2 (IL-4, IL-10, IL-6 and TGF-β) cytokines were not obviously changed. However, IL-6 has complex relationships with IL-17^23^ and declined slightly but not significantly. COX2 has an important role in the implantation process^45^ and was also changed following IL-17A blockade. A similar situation was observed with the expression of MMP2 and MMP9, which are essential for implantation and decidualization^46^,^47^. Overall, blocking IL-17A does not alter the Th1/Th2 balance, but rather, regulates inflammation by regulating the expression of various chemokines and cytokines.

**Figure 5:**
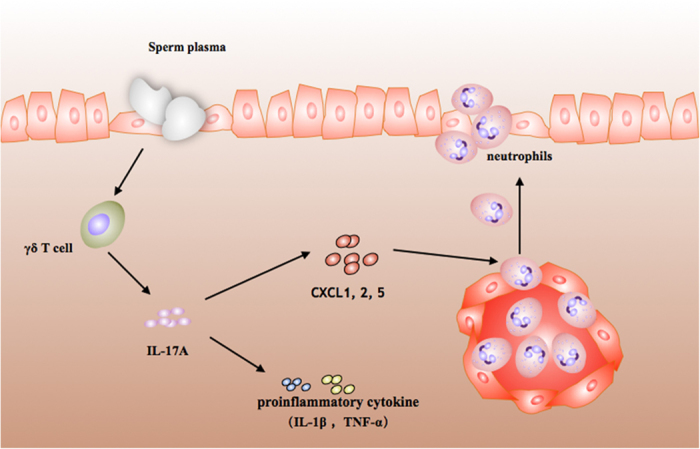
Seminal plasma regulates uterine inflammation through the γδ T cell/IL-17A axis. Our result indicated that seminal plasma could stimulate the γδ T cells to secrete IL-17A. While IL-17A can regulate the chemokines CXCL1, CXCL2 and CXCL5 to promote the recruitment of neutrophils, it can also regulate pro-inflammatory cytokines (IL-1β and TNF-α), both of which promote inflammation in the uterus.

Inflammation could prepare the uterus for successful implantation^6^ and our work confirmed that seminal plasma could stimulated uterine inflammation through the γδ T cells/IL-17A axis. We also found that IL-17A induced cytokines (IL-1β and TNF-α) and chemokines (CXCL1, CXCL2, CXCL5 and CCL20) to promote the recruitment of neutrophils (Fig. 5). However, more research needs to focus on the molecular mechanism of γδ T cells stimulation and how the level of inflammation influences implantation and decidualization.

## Methods

### Mice

Sexually mature BALB/c mice (10–12 weeks old) were purchased from SPF Laboratory Animal Technology (Beijing, China). The mice were housed in a temperature- and humidity-controlled, pathogen-free facility with a 12 hour light-dark cycle (12 L: 12 D). The Institutional Animal Care and Use Committee of the Institute of Zoology, Chinese Academy of Science approved all the procedures. All experiments were performed in accordance with the Institutional Animal Care and Use Committee guidelines. The female male mice were caged with fertile or infertile males at a 2:1 ratio, and the presence of a vaginal plug was designated as day 0.5 (D0.5) of pregnancy. For infertile males, vasectomy was performed in BALB/c male mice under anaesthesia.

### Real-time PCR

Total RNA was extracted using an RNA isolation kit (Bio Teke, Beijing, China). The RNA templates were then reverse-transcribed into cDNA (Promega, Madison WI, USA). The cDNA was amplified with SYBR Green Master Mix reagents (ComWin Biotech Co., Ltd., Beijing, China) by a two-step real-time PCR reaction performed on a LightCycler 480 (Roche, Indianapolis, IN, USA). In brief, the cDNA templates were heated to 95 °C for 10 min. Then, a forty-five-cycle reaction was performed that included denaturation at 95 °C for 15 s and extension at 60 °C for 1 min. The primers that were used are summarized in Supplementary Table 1. The ΔΔCt method, which normalizes target gene mRNA expression to GAPDH expression, was used to quantify the mRNA expression of target genes.

### Reagents

For IL-17A neutralization, an anti-IL-17A antibody (50104; R&D Systems, Minneapolis, MN, USA) or an isotype control (54447; R&D Systems) was administered by intravenous injection (200 μg/kg)^48^ on D0.5. The antibody was dissolved in 100 μL of saline. The mice were euthanized on D1, and their uteri were excised for analysis.

### Immunohistochemistry

Frozen section (8 μm) of mouse uterine tissue was fixed in pre-cold cooled methanol for 10 min., Then, after the tissue was incubated with 0.3% H_2_O_2_ for 10 min at room temperature, and the slides were blocked in 5% bovine serum albumin (BSA) (Invitrogen, CA) for 1 h at 37 °C. Next, the sections were incubated with anti-Ly-6G (RB6-8C5; eBiosciences, San Diego, CA, USA) diluted in PBS (1:500) overnight at 4 °C. Then, the sections were incubated with a secondary antibody conjugated to HRP at 37 °C for 1 h. Finally, the slides were stained with diaminobenzidine and counterstained with haematoxylin, and then observed and photographed using a Nikon H600L (Japan) microscope.

### Enzyme-linked immunosorbent assays (ELISA)

Uterine tissue was ground to fine powder in liquid nitrogen. Then, the homogenized biopsies were weighted and lysed using a non-denaturing lysis buffer (EDTA free, Applygen, Beijing, China) on ice. Next, the total protein lysate was centrifuged 15min for 15000 × g at 4 °C. The supernatants were analyzed with Mouse IL-17A (homodimer) ELISA kit (eBioscience), according to the manufacturer’s instructions.The optical density was measured using a Bio-Rad 3550 micro-plate reader(Bio-Rad, Hercules, CA) at 450 nm.

### Tissue preparation

After core needle grinding, the splenocytes were lysed using an ammonium chloride lysing solution, which lysed the blood cells directly. Uterine cells were prepared according to the protocol previously described^49^. Briefly, uteri that were minced into small fragments were incubated with 200 U/mL of hyaluronidase (Sigma-Aldrich, St. Louis, MO, USA), 1 mg/mL collagenase type IV (Sigma-Aldrich) and 0.2 mg/mL DNase (Sigma-Aldrich) in HBSS/Ca/Mg at 37 °C for 20 min. During this incubation, the tissues were pipetted up and down every 5 min, and the reaction was terminated with PBS containing 0.2% BSA. Cauda epididymal spermatozoa from male BALB/c mice were collected into PBS and cultured at 37 °C with 5% CO_2_ for 20 min. The samples were then centrifuged at 300 × *g* for 5 min and incubated in RPMI-1640 medium (HyClone, Logan, UT, USA) supplemented with 10% foetal bovine serum (FBS) (HyClone) and penicillin/streptomycin (100 U/mL) (1 × 10^6^ cells/mL). Seminal plasma was collected from female mice that had mated with a vasectomized male on D0.5.

### Cytokine stimulation

Uteri from virgin mice (on the day of estrogen) were dissected free from the mesometrium and removed the cervix. The cells were processed as described in the tissue preparation section of the methods (under sterile conditions). The cells were incubated in 500 μL of RPMI-1640 media supplemented with 10% FBS and penicillin/streptomycin at 37 °C and 5% CO_2_ overnight. On the next day, the uterine cells co-cultured with suspension were co-cultured with different stimulators in a 24-well plate (3 × 10^5^ cells/well). The stimulators that were used included: a. control: brefeldin A (50 ng/mL), b. positive control: 2 μl/mL of Cell Stimulation Cocktail (eBioscience), c. sperm cell: sperm cells (1:1 with uterine tissue cells) and brefeldin A (50 ng/mL) (Selleckchem, Houston, TX, USA), and d. seminal plasma: 0.5% seminal plasma and brefeldin A (50 ng/mL). The cells were incubated in 500 μL of RPMI-1640 media supplemented with 10% FBS and penicillin/streptomycin at 37 °C and 5% CO_2_ for 8 h.

### Flow cytometry

The cells were processed as described in the tissue preparation section of the methods. After processing, the cells suspensions were blocked with a CD16/32 antibody for 10 min at 4 °C and stained with fluorochrome labelled antibodies at 4 °C for 30 min. After the staining was performed, the cell were rinsed with PBS containing 0.2% BSA for analysis. For intracellular antigen staining, the Foxp3/Transcription Factor Staining Buffer Set (00-5523, eBioscience) was used according to the manufacturer’s protocol after the surface staining was complete. Peridinin chlorophyll protein (PerCP) Cyanine5.5-conjugated anti-CD45 (30-F11), phycoerythrin (PE)-conjugated anti-CD3e (145-2C11), fluorescein isothiocyanate (FITC)-conjugated anti-γδTCR (GL3), allophycocyanin (APC)-conjugated anti-IL-17A (eBio17B7), and Ly-6G (RB6-8C5) were purchased from eBiosciences. The samples were analysed using a FACScalibur or were sorted using a FACSAria (BD Biosciences, Franklin Lakes, NJ, USA). The samples were analysed using FlowJo software 10.6 (Tree Star, Inc.).

### Statistics

Statistical analyses were performed using SPSS version 20.0 software (SPSS, Chicago, IL, USA). The data are reported as the mean ± SEM. A p value of less than 0.05 (*) represents a significant difference, and a p value of less than 0.01 (**) represents a highly significant difference.

## Additional Information

**How to cite this article**: Song, Z.-H. *et al.* Seminal plasma induces inflammation in the uterus through the γδ T/IL-17 pathway. *Sci. Rep.* **6**, 25118; doi: 10.1038/srep25118 (2016).

## Supplementary Material

Supplementary Information
