# Supplementary material for: Seminal plasma induces inflammation in the uterus through the γδ T/IL-17 pathway
Source: Sci Rep. 2016 Apr 25;6:25118. doi: 10.1038/srep25118 (PMC4842971; doi:10.1038/srep25118)
Supplement: Supplementary Information [file srep25118-s1.pdf]

Title

Seminal plasma induces inflammation in the uterus through the γδ T/IL-17 pathway

Authors

Zhi-Hui Song,[1,2] Zhong-Yin Li,[1] Dan-Dan Li,[1,2] Wen-Ning Fang,[1,2] Hai-Yan Liu,[1] Chao-Yang Meng,[1,2] Dan-Dan Yang,[1,2] Ying Yang,[1] and Jing-Pian Peng[*,1]

Affiliations

[1] State Key Laboratory of Stem Cell and Reproductive Biology, Institute of Zoology, Chinese Academy of Sciences, Beijing, P.R. China; and [2] University of Chinese Academy of Sciences, Beijing, P.R. China

[*]Corresponding author: State Key Laboratory of Stem Cell and Reproductive Biology, Institute of Zoology, Chinese Academy of Sciences, Beijing, P.R. China. Tel: 86-10-64807183; Fax: 86-10-64807099; E-mail address: pengjp@ioz.ac.cn

Gene		Primer Squence
**IL-17A**	forward	5’-GGCCCTCAGACTACCTCAAC-3’
	reverse	5’-TCTCGACCCTGAAAGTGAAGG-3’
**CXCL1**	forward	5’-ACTGCACCCAAACCGAAGTC-3’
	reverse	5’-TGGGGACACCTTTTAGCATCTT-3’
**CXCL2**	forward	5’-CCAACCACCAGGCTACAGG-3’
	reverse	5’-GCGTCACACTCAAGCTCTG-3’
**CXCL5**	forward	5’-TGCGTTGTGTTTGCTTAACCG-3’
	reverse	5’-CTTCCACCGTAGGGCACTG-3’
**CCL20**	forward	5’-ACTGTTGCCTCTCGTACATACA-3’
	reverse	5’-GAGGAGGTTCACAGCCCTTTT-3’
**MMP2**	forward	5’-ACCTGAACACTTTCTATGGCTG-3’
	reverse	5’-CTTCCGCATGGTCTCGATG-3’
**MMP9**	forward	5’-GCAGAGGCATACTTGTACCG-3’
	reverse	5’-TGATGTTATGATGGTCCCACTTG-3’
**IL-1β**	forward	5’-GGACAGAATATCAACCAACAAGTGATA-3’
	reverse	5’-GTGTGCCGTCTTTCATTACACAG-3’
**IL-4**	forward	5’-GGTCTCAACCCCCAGCTAGT-3’
	reverse	5’-GCCGATGATCTCTCTCAAGTGAT-3’
**IL-6**	forward	5’-AACCACGGCCTTCCCTACT-3’
	reverse	5’-CATTTCCACGATTTCCCAGA-3’

**IL-10**	forward	5’-CGCAGCTCTAGGAGCATGTG-3’
	reverse	5’-AGGAGCTGTCATTAGGGACATC-3’
**COX2**	forward	5’-TGAGCAACTATTCCAAACCAGC-3’
	reverse	5’-GCACGTAGTCTTCGATCACTATC-3’
**TGF-β**	forward	5’-CTTCAATACGTCAGACATTCGGG-3’
	reverse	5’-GTAACGCCAGGAATTGTTGCTA-3’
**TNF-α**	forward	5’-CTGAACTTCGGGGTGATCGG-3’
	reverse	5’-GGCTTGTCACTCGAATTTTGAGA-3’
**IFN-γ**	forward	5’-ACAGCAAGGCGAAAAAGGATG-3’
	reverse	5’-TGGTGGACCACTCGGATGA-3’
**GAPDH**	forward	5’-AGGTCGGTGTGAACGGATTTG-3’
	reverse	5’-GGGGTCGTTGATGGCAACA-3’

Supplementary Table 1. Primers for the detection of mRNA expression by Real-time PCR.

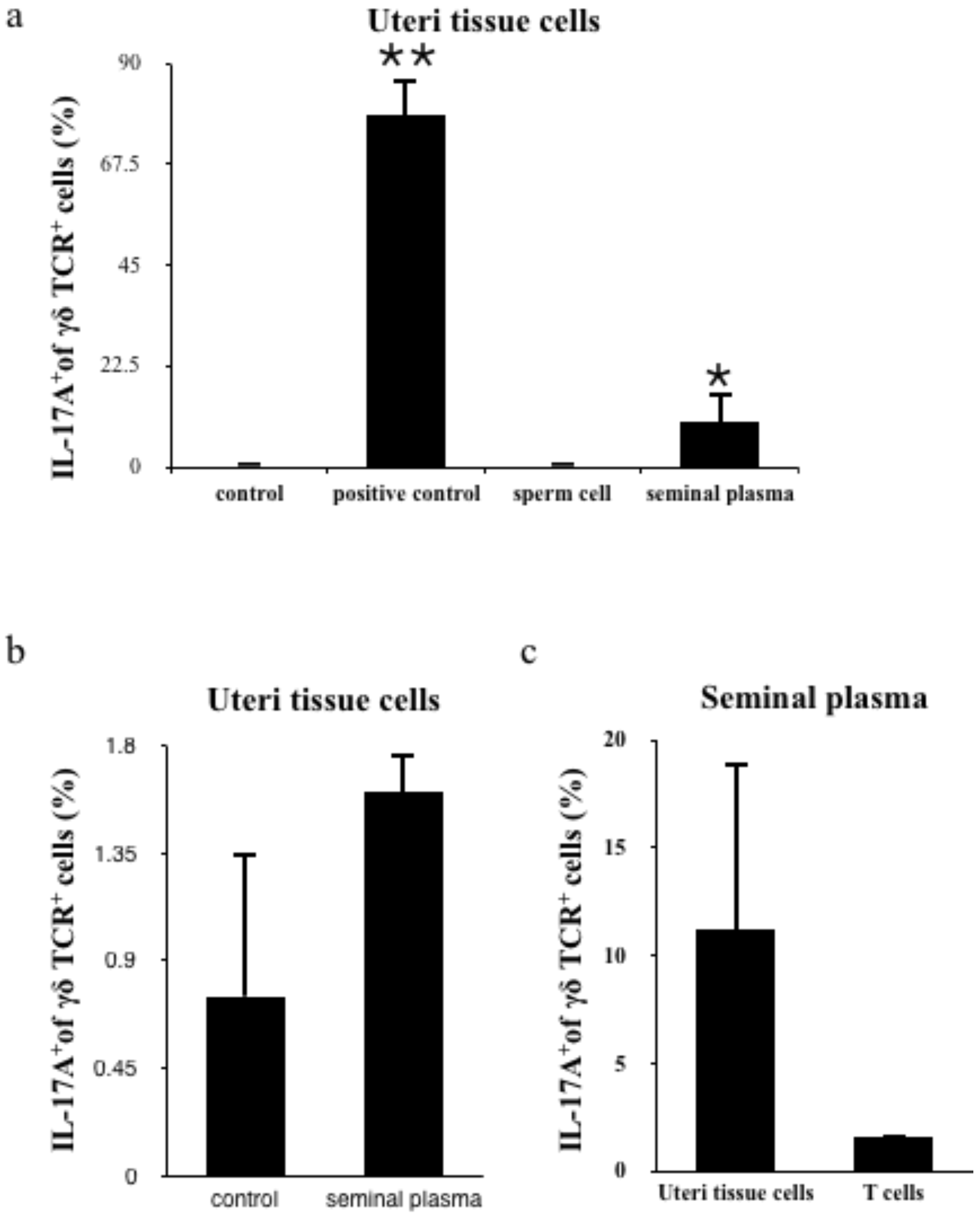

Supplementary Figures 1. Analysis for percentage of IL-17A positive proportion in γδ T cells. (a) and (b) statistical analysis for figure 2e and f. Data show the mean ± S.E.M summary of four (figure 2e) or three (figure 2f) independent experiments with independent samples T-test (c) Uterine tissue cells and T cells sorted from uterine tissue cells stimulated with the 0.5% seminal plasma. Data show the mean ± S.E.M summary of four (uteri tissue cells) or three (T cells) independent experiments with independent samples T-test (P=0.076).

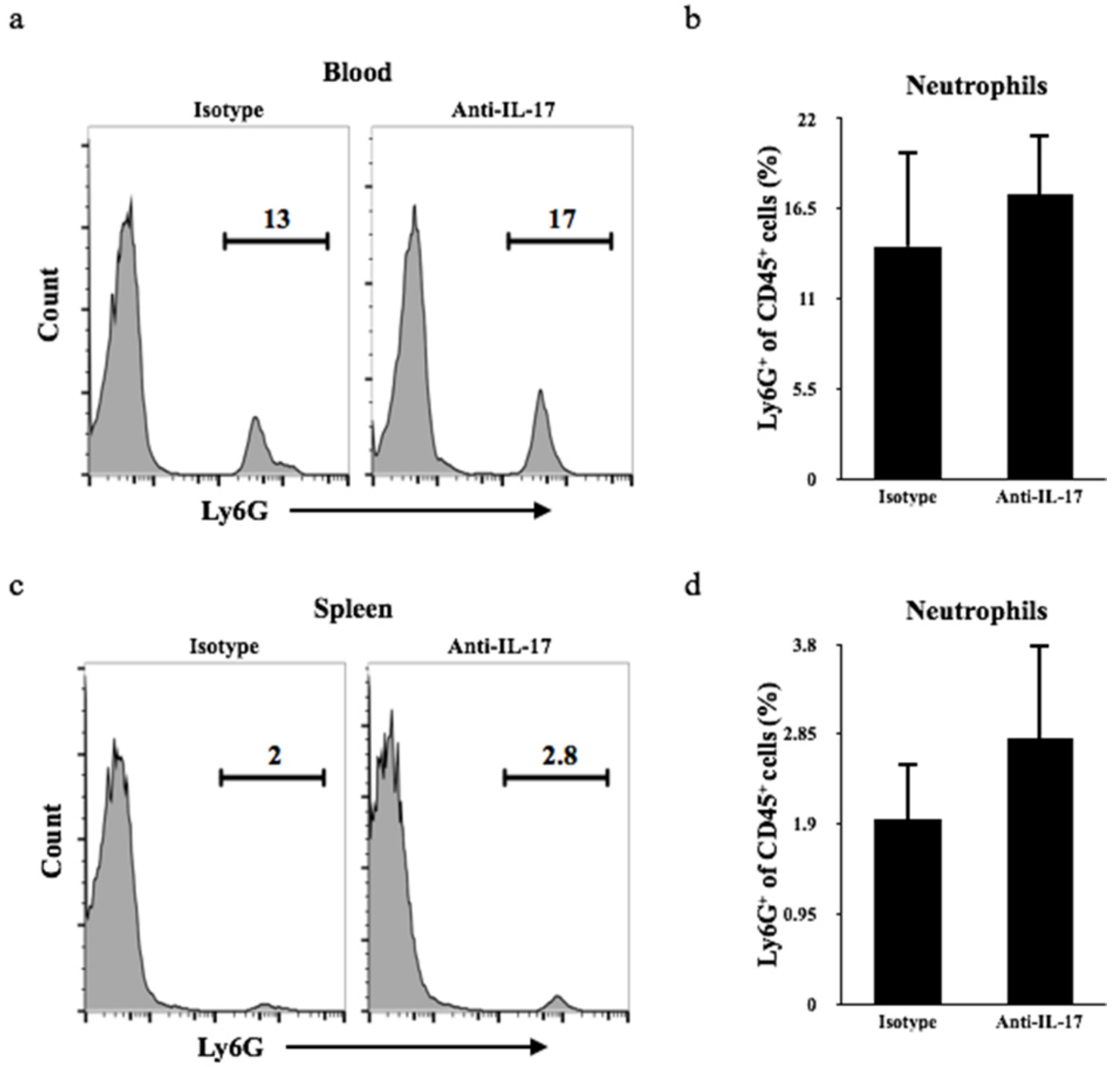

Supplementary Figures 2. IL-17A blocking did not changes the percentage of neutrophils in blood and spleen. (a) and (b) Percentage of neutrophils in the CD45 gated population in blood， Numbers represent the percentage of population within the indicated gates. (c) and (d) Percentage of neutrophils in the CD45 gated population in spleen Numbers represent the percentage of population within the indicated gates. Data show the mean ± S.E.M summary of four independent experiments with independent samples T-test.
